# Beyond the Knot: A Pragmatic Comparison of Monocryl and Prolene for Neurosurgical Wound Closure

**DOI:** 10.7759/cureus.112139

**Published:** 2026-07-06

**Authors:** Muhammad Shakir, Khawar Zaman, Rabia Saleem, Talha Sajid, Muhammad A Zafar, Muhammad Arslan, Abdul Majid

**Affiliations:** 1 Neurological Surgery, Punjab Institute of Neurosciences, Lahore, PAK

**Keywords:** neurosurgical procedures, polypropylenes, surgical wound infection, sutures, wound closure techniques

## Abstract

Background

Surgical site infection (SSI) is a major source of morbidity and additional cost following neurosurgical procedures. Although absorbable monofilament sutures (poliglecaprone 25 and Monocryl) and non-absorbable polypropylene sutures (Prolene) are both routinely used for skin closure, direct comparative data in mixed cranial and spinal neurosurgical cohorts remain limited, particularly from low- and middle-income settings. We compared Monocryl and Prolene for neurosurgical skin closure in a descriptive (nonequivalence) design. The primary outcome was SSI; secondary outcomes were skin closure time, length of hospital stay, and inpatient postoperative hospitalization cost.

Methods

This single-center retrospective cohort study included 523 adult patients (≥18 years) undergoing elective or emergency cranial or spinal neurosurgical procedures at a public-sector tertiary neurosciences institute between January 2024 and December 2025. Patients were grouped by skin closure material (Monocryl or Prolene). SSI was defined according to the United States CDC 2017 guideline. Continuous variables were compared using the Mann-Whitney U test and reported as mean ± SD and median with IQR; categorical variables were compared using the chi-square test or Fisher’s exact test. Multivariable logistic regression was used to identify independent predictors of SSI. A two-sided p-value <0.05 was considered statistically significant.

Results

Of 523 patients (mean age 46.3 ± 16.6 years; 51.4% male), 221 (42.3%) underwent skin closure with Monocryl and 302 (57.7%) with Prolene. The overall incidence of SSI was 12.0%. SSI was numerically lower in the Monocryl group (9.5%) than in the Prolene group (13.9%), but this difference did not reach statistical significance (p = 0.126). There were no significant between-group differences in CSF leak, reoperation, or readmission rates. Skin closure time was significantly shorter with Monocryl than with Prolene (median 7 minutes, IQR 6-9 vs median 14 minutes, IQR 12-17; p < 0.001). Length of hospital stay (p = 0.125) and inpatient hospitalization cost (p = 0.170) did not differ significantly between groups. Multivariable logistic regression did not identify any independent predictors of SSI (model omnibus p = 0.136). The two groups differed significantly in procedure type (elective vs emergency; p < 0.001), indicating confounding by indication.

Conclusions

In this large neurosurgical cohort, we did not detect a statistically significant difference in SSI or other adverse postoperative outcomes between Monocryl and Prolene; however, the study was not designed or powered to establish equivalence, and the two groups were imbalanced by procedure type. Monocryl was associated with significantly faster skin closure, although this comparison also reflects a difference in closure technique between groups. These findings should be regarded as hypothesis-generating and warrant confirmation in adequately powered, indication-matched prospective studies.

## Introduction

Surgical site infection (SSI) remains one of the most clinically and economically important complications in modern neurosurgical practice. It contributes to increased morbidity and mortality, prolonged hospitalization, repeat surgery, and substantial additional healthcare costs [1,2]. Reported SSI rates following neurosurgical procedures vary widely depending on the type of surgery, patient comorbidity profile, and perioperative care, with most contemporary cohorts reporting rates between 1% and 8% for cranial procedures and 1-15% for spinal procedures [3-6]. Cranial procedures carry distinct risks related to dural breach, CSF leak, and meningitis, while spinal surgery, particularly with instrumentation, is associated with deep wound and implant-related infections [3,7,8].

Skin closure is the final and arguably most patient-visible step of any neurosurgical procedure. Beyond aesthetic outcome, the integrity of skin closure provides the first mechanical barrier against microbial invasion of the deeper surgical bed [9]. The choice of skin closure material is therefore not trivial: it influences the local wound microenvironment, the tissue reactivity and biofilm formation potential of the implanted suture, the speed of wound closure, and the need for subsequent suture removal, all of which bear on infection risk, operative efficiency, and patient experience [9]. A wide range of skin closure materials and techniques is in routine clinical use, including absorbable monofilament sutures, non-absorbable monofilament sutures, surgical staples, and tissue adhesives [10-13]. Among sutures, absorbable monofilament poliglecaprone 25 (Monocryl) and non-absorbable polypropylene (Prolene) are two of the most commonly employed materials in everyday neurosurgical practice.

Existing comparative literature on absorbable vs non-absorbable sutures, drawn largely from general, plastic, and orthopedic surgical settings, suggests broadly comparable rates of wound infection between the two material classes, with potential advantages of absorbable sutures in operative efficiency, patient comfort, and the avoidance of suture removal visits [9,14,15]. A randomized controlled trial comparing Monocryl with nylon sutures in carpal tunnel surgery similarly demonstrated comparable safety with improved early patient- and observer-reported scar outcomes for the absorbable arm [9]. However, direct comparative evidence specifically evaluating Monocryl and Prolene in neurosurgical populations, and especially in cohorts that combine both cranial and spinal procedures, remains scarce [4,11].

The relevance of this knowledge gap is amplified in low- and middle-income healthcare settings, where resource utilization, the cost of consumables, and surgical workload exert a substantial influence on clinical decision-making [16,17]. SSI imposes a disproportionate economic burden in such settings, where reported neurosurgical and general-surgical infection rates are frequently higher than in high-income countries and the marginal cost of each additional inpatient day is keenly felt [16]. In high-volume public-sector centers, even small reductions in skin closure time or length of postoperative stay can translate into meaningful gains in operating-room throughput, bed availability, and total cost of care [16-18]. Despite this, comparative neurosurgical data from South Asia and similar resource-constrained settings are sparse, and most existing studies focus narrowly on infection rates without simultaneously evaluating operative efficiency, length of stay, or cost.

We therefore designed this single-center retrospective cohort study to compare Monocryl and Prolene for neurosurgical skin closure in a mixed cranial and spinal cohort. This was a descriptive comparative study and was not designed as an equivalence or non-inferiority trial. The primary objective was to compare the incidence of SSI between patients receiving Monocryl vs Prolene skin closure. The secondary objectives were to compare skin closure time, length of postoperative hospital stay, and inpatient postoperative hospitalization cost between the two groups and to identify independent predictors of SSI using multivariable analysis.

## Materials and methods

Study design and setting

This was a retrospective cohort study conducted in the Department of Neurosurgery at the Punjab Institute of Neurosciences (PINS), Lahore, Pakistan, a high-volume, public-sector, tertiary care neurosciences referral center. We included consecutive adult patients who underwent cranial or spinal neurosurgical procedures with primary skin closure between January 2024 and December 2025 (a 24-month consecutive enrollment window). For the last-enrolled patients, the minimum 30-day surveillance period (90 days for implant procedures) was completed within the first quarter of 2026, after which the dataset was locked, and the analysis, manuscript preparation, and submission were carried out during 2026. The study was reported in accordance with the Strengthening the Reporting of Observational Studies in Epidemiology (STROBE) guideline for cohort studies.

Inclusion and exclusion criteria

Eligible patients were adults aged 18 years or older who underwent elective or emergency cranial or spinal neurosurgical procedures at our institution during the study period and in whom skin closure was performed using either Monocryl or Prolene as the principal skin closure material. Patients were excluded if they had incomplete medical records (defined as missing data on the exposure or any of the four primary outcome variables), if skin closure had been performed using alternative materials such as surgical staples or tissue adhesives, or if they were lost to follow-up before completion of the minimum 30-day surveillance period. Of the records screened during the study period, complete data on the exposure and all outcomes were available for the 523 patients included; cases with incomplete documentation or follow-up shorter than 30 days were excluded prior to analysis.

Skin closure technique

Patients were grouped according to the principal material used for definitive skin-layer closure: Monocryl (poliglecaprone 25, absorbable monofilament) or Prolene (polypropylene, non-absorbable monofilament). In keeping with departmental practice, Monocryl was most often placed as a continuous subcuticular suture, whereas Prolene was most often placed as an interrupted transcutaneous suture; however, the suture caliber (commonly 2-0 to 3-0) and the precise closure technique were ultimately at the discretion of the operating surgeon and varied with incision length, anatomical site, and patient factors. The procedure-level distribution of interrupted vs continuous technique and of suture caliber was not captured in a structured field in the medical record and could not be reliably reconstructed retrospectively; this is acknowledged as a limitation. In routine clinical practice at our institution, Monocryl was more commonly chosen for elective procedures and Prolene for emergency procedures, though both materials were used across both contexts. All procedures were performed within a single neurosurgical department by the institution’s neurosurgical teams (consultant neurosurgeons together with supervised residents); the number and identity of the individual operating surgeons were not recorded in a structured, analyzable field, and surgeon-level clustering could, therefore, not be modeled. Subgaleal and deeper-layer closure was standardized between groups in accordance with departmental practice.

Data collection and variables

Data were extracted retrospectively from inpatient medical records, operative notes, anesthesia charts, and postoperative follow-up documentation by members of the neurosurgical team using a standardized data-collection proforma. Demographic variables included age and sex. Clinical variables included relevant comorbidities (diabetes mellitus, obesity defined as BMI >30 kg/m², malnutrition, and combinations thereof). Procedural variables included the type of procedure (elective vs emergency), the anatomical category of surgery (cranial vs spinal), and the use of any operative implant (e.g., aneurysm clips, miniplates, and cranioplasty material).

The principal exposure was the type of skin closure material (Monocryl or Prolene). The prespecified primary outcome was SSI. The prespecified secondary outcomes were (i) estimated skin closure time in minutes, recorded from the documented start and end of skin-layer closure on the anesthesia or operative record; (ii) length of postoperative hospital stay in days; and (iii) inpatient postoperative hospitalization cost in Pakistani rupees (PKR). CSF leak, reoperation, and readmission were recorded as additional safety outcomes. SSI was defined according to the United States CDC 2017 guideline as any infection involving the superficial incisional, deep incisional, or organ/space tissue planes within 30 days of the index procedure (or within 90 days for procedures involving an implant) [19,20]. Skin closure time was derived from the timed operative and anesthesia records; because this relied on routinely documented intraoperative timings rather than a dedicated stopwatch protocol, some measurement imprecision is possible and is acknowledged as a limitation. A CSF leak was defined as the clinical or radiological identification of CSF extravasation through the surgical wound. Reoperation referred to any unplanned return to the operating theater related to the index procedure during the same admission or follow-up period. Readmission referred to any unplanned hospital readmission within 30 days of discharge that was attributable to the index surgery.

Cost calculation

Cost analysis was performed from the public-sector hospital perspective and was confined to inpatient postoperative care. The inpatient hospitalization cost per patient was estimated by multiplying the institutional average daily cost of inpatient ward care (covering bed occupancy, nursing services, routine medications, and standard postoperative management) by the patient’s documented length of stay. Operative theater time, anesthesia services, surgical consumables, and the unit cost of suture material itself were not separately itemized in this analysis, as these are not routinely captured on a per-patient basis in our institutional accounting framework. Accordingly, the cost outcome reported should be interpreted as a length-of-stay-driven inpatient cost proxy rather than a comprehensive total cost of care.

Statistical analysis

All analyses were performed using IBM SPSS Statistics for Windows, version 26.0 (released 2018; IBM Corp., Armonk, NY, USA). Continuous variables were tested for normality with the Shapiro-Wilk test and were found to be non-normally distributed (p < 0.05 for all four continuous variables). Continuous variables are therefore reported as both mean ± SD and median with IQR and were compared between groups using the nonparametric Mann-Whitney U test. Categorical variables are reported as frequencies and percentages and were compared using the chi-square test, with Fisher’s exact test substituted where any expected cell count was less than five. Multivariable logistic regression was used to identify independent predictors of SSI; covariates included age, sex, comorbidities, procedure type (elective vs emergency), surgery category (cranial vs spinal), implant use, skin closure material, and estimated skin closure time. Model fit was assessed using the Omnibus chi-square test, the Hosmer-Lemeshow goodness-of-fit test, and Nagelkerke’s R². A two-sided p-value of less than 0.05 was considered statistically significant. No propensity-score matching or formal sensitivity analyses were performed.

As this was a retrospective analysis of a fixed consecutive cohort, no a priori sample-size calculation was performed. To contextualize the precision of the primary comparison, a post hoc power analysis was conducted for the SSI outcome. Given the observed proportions (Monocryl 9.5% vs Prolene 13.9%) at a two-sided alpha of 0.05, the available samples (221 vs 302) provided approximately 33% statistical power to detect a difference of the observed magnitude. Detecting such a difference at 80% power would have required approximately 850 patients per group, and detecting a 30% relative reduction in SSI would have required approximately 940 patients per group. The study was therefore underpowered for the primary outcome, and the SSI comparison should be interpreted accordingly.

Ethical considerations

The study was reviewed by the Institutional Review Board (IRB) of the Punjab Institute of Neurosciences (approval 2506/IRB/PINS/Approval/2026; dated February 6, 2026) and was determined to be exempt from formal IRB approval, as it presented no harm to the participants studied. The requirement for written informed consent was waived in view of the retrospective design. Patient confidentiality was strictly maintained throughout the study, and all data were anonymized before analysis.

## Results

A total of 523 patients undergoing cranial or spinal neurosurgical procedures with primary skin closure during the study period met the inclusion criteria and were included in the final analysis. The cohort comprised 269 males (51.4%) and 254 females (48.6%), with a mean age of 46.3 ± 16.6 years (median 46, IQR 33-59). Elective procedures accounted for 309 cases (59.1%), and cranial surgeries comprised 335 cases (64.1%) of the total. Skin closure was performed using Monocryl in 221 patients (42.3%) and using Prolene in 302 patients (57.7%). The overall incidence of SSI was 12.0%, while CSF leak, reoperation, and readmission occurred in 5.2%, 1.0%, and 6.5% of patients, respectively. Baseline characteristics of the cohort, stratified by skin closure material, are presented in Table 1. The two groups were broadly comparable in age, sex distribution, comorbidity burden, surgery category, and implant use; however, procedure type differed significantly between groups, with a greater proportion of elective cases in the Monocryl arm and a correspondingly greater proportion of emergency cases in the Prolene arm (p < 0.001). Continuous outcome variables were non-normally distributed on Shapiro-Wilk testing, and nonparametric tests were therefore used for between-group comparisons.

**Table 1:** Baseline demographic, clinical, and procedural characteristics stratified by skin closure material (n = 523) Continuous variables were compared using the Mann-Whitney U test (U-value column); categorical variables were compared using the chi-square test (χ² column reports test statistic and degrees of freedom), with Fisher’s exact test substituted where any expected cell count was less than 5. “Combined comorbidities” refers to patients with more than one of diabetes, obesity, or malnutrition. “Implant used (yes)” includes any operative implant such as aneurysm clips, miniplates, or cranioplasty material.

Variable	Monocryl (n = 221)	Prolene (n = 302)	U-value	χ² (df)	p-value
Continuous variables					
Age (years), mean ± SD	46.0 ± 16.4	46.5 ± 16.7	32,795.70	-	0.736
Age (years), median (IQR)	46 (33-59)	46 (33-59)	-	-	-
Categorical variables, n (%)					
Sex: male	113 (51.1)	156 (51.7)	-	0.001 (1)	0.976
Sex: female	108 (48.9)	146 (48.3)	-	-	-
Comorbidities: none	187 (84.6)	250 (82.8)	-	0.423 (4)	0.981
Comorbidities: diabetes mellitus	20 (9.0)	29 (9.6)	-	-	-
Comorbidities: obesity (BMI > 30 kg/m²)	7 (3.2)	11 (3.6)	-	-	-
Comorbidities: malnutrition	6 (2.7)	10 (3.3)	-	-	-
Comorbidities: combined	1 (0.5)	2 (0.7)	-	-	-
Procedure type: elective	166 (75.1)	143 (47.4)	-	39.545 (1)	<0.001
Procedure type: emergency	55 (24.9)	159 (52.6)	-	-	-
Surgery category: cranial	145 (65.6)	190 (62.9)	-	0.295 (1)	0.587
Surgery category: spinal	76 (34.4)	112 (37.1)	-	-	-
Implant used: yes (any type)	65 (29.4)	94 (31.1)	-	0.105 (1)	0.745
Implant used: no	156 (70.6)	208 (68.9)	-	-	-

Postoperative outcomes and operative efficiency by skin closure material

**Figure 1:**
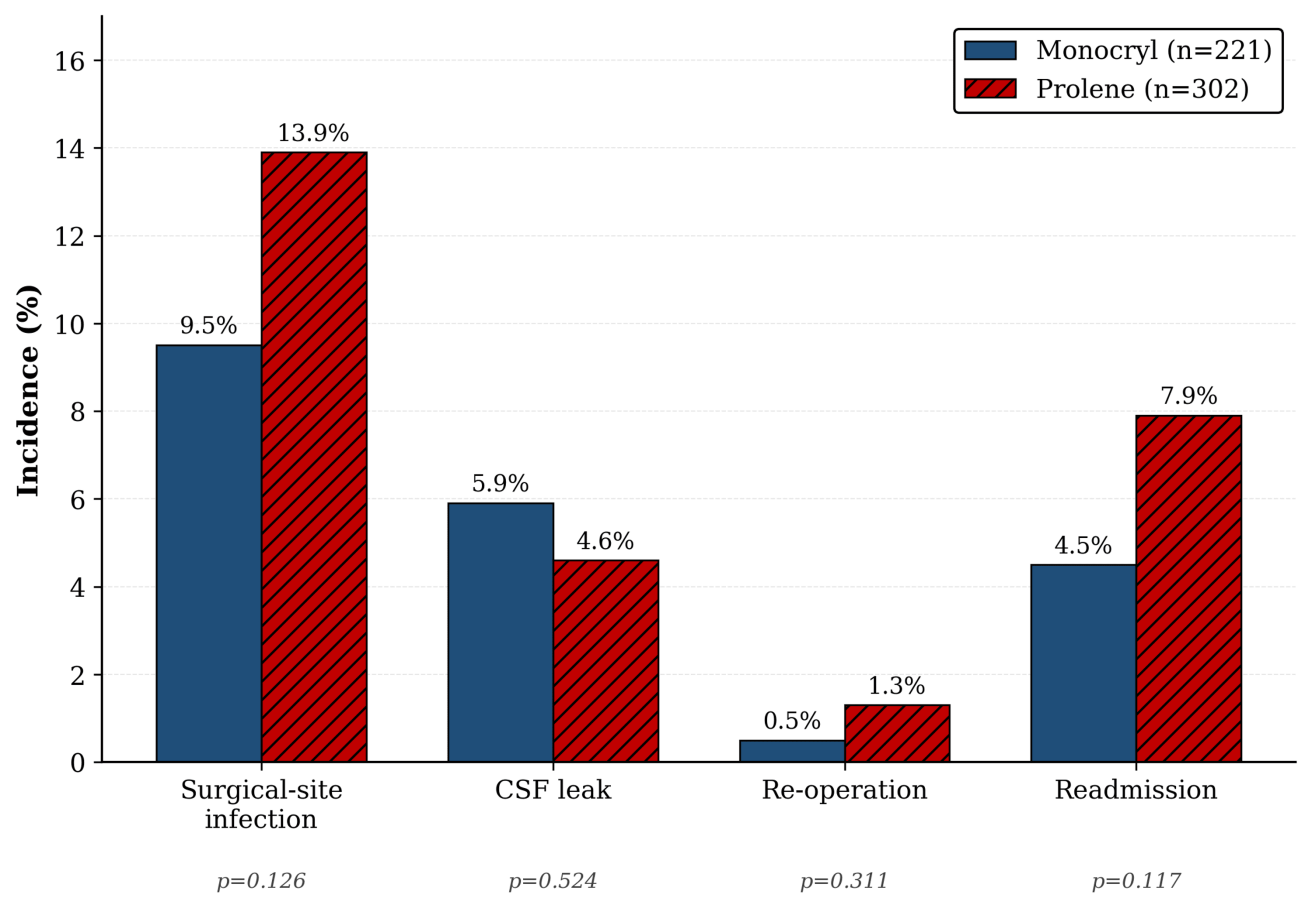
Postoperative categorical outcomes by skin closure material Side-by-side comparison of incidence rates (%) for SSI, CSF leak, reoperation, and readmission in the Monocryl (n = 221) and Prolene (n = 302) groups. None of the between-group differences reached statistical significance on chi-square testing: SSI χ² = 2.337, df = 1, p = 0.126; CSF leak χ² = 0.405, df = 1, p = 0.524; reoperation χ² = 1.025, df = 1, p = 0.311; readmission χ² = 2.459, df = 1, p = 0.117 df, degrees of freedom; SSI, surgical site infection

**Figure 2:**
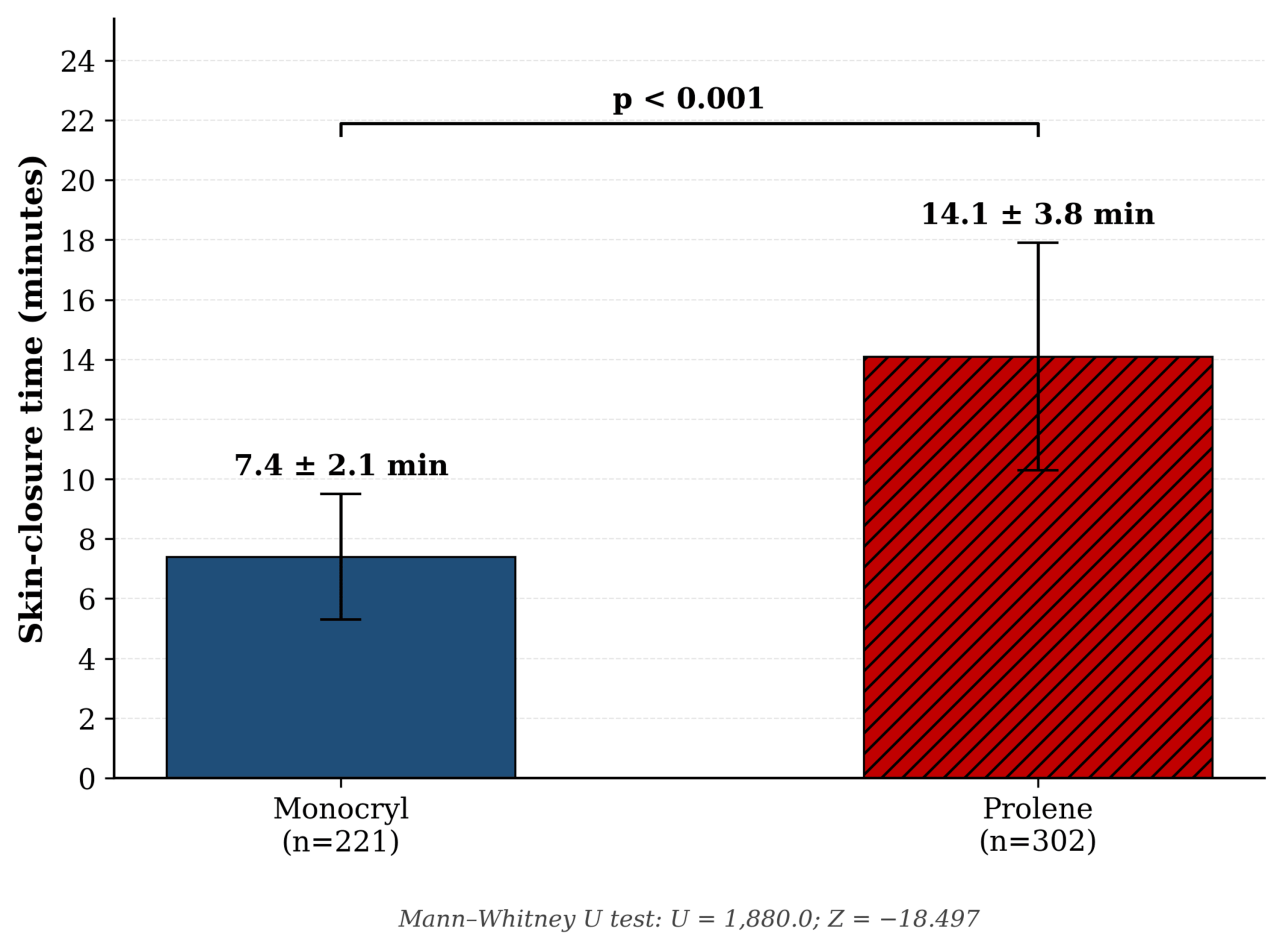
Estimated skin closure time by suture material Mean estimated skin closure time (minutes) for the Monocryl and Prolene groups, with error bars representing one SD. Skin closure was significantly faster with Monocryl than with Prolene on Mann-Whitney U testing (p < 0.001). This comparison reflects a combined difference in both suture material and closure technique, as Monocryl was predominantly placed as a continuous subcuticular suture and Prolene as an interrupted transcutaneous suture; the observed time difference should therefore not be attributed to the material alone.

The incidence of SSI was lower in the Monocryl group (21/221, 9.5%) than in the Prolene group (42/302, 13.9%), but this difference did not reach statistical significance (χ² = 2.337, df = 1, p = 0.126). There were no statistically significant between-group differences in the rates of CSF leak (5.9% vs 4.6%, p = 0.524), reoperation (0.5% vs 1.3%, p = 0.311), or readmission (4.5% vs 7.9%, p = 0.117) (Figure 1). For continuous outcomes, skin closure time was significantly shorter in the Monocryl group than in the Prolene group, with a large effect size (median 7 minutes, IQR 6-9 vs median 14 minutes, IQR 12-17; mean ranks 119.5 vs 366.3; U = 1,880.0; Z = −18.497; p < 0.001) (Figure 2). In contrast, there were no statistically significant differences between groups in length of hospital stay (median 4, IQR 3-6 days vs median 4, IQR 3-6 days; mean ranks 273.7 vs 253.5; U = 30,790.5; Z = −1.533; p = 0.125) or in inpatient hospitalization cost (median PKR 18,000 vs PKR 18,000; mean ranks 270.8 vs 255.6; U = 31,425.0; Z = −1.372; p = 0.170). A complete comparison of postoperative categorical outcomes and continuous variables by skin closure material is presented in Table 2.

**Table 2:** Postoperative categorical outcomes and continuous variables by skin closure material The chi-square test was used for categorical outcomes; Fisher’s exact test was substituted where any expected cell count was less than 5. The Mann-Whitney U test was used for continuous variables due to non-normal distribution on Shapiro-Wilk testing. df, degrees of freedom; PKR, Pakistani Rupees; SSI, surgical site infection

Outcome	Monocryl (n = 221)	Prolene (n = 302)	Test statistic	p-value
Categorical outcomes, n (%)				
SSI	21 (9.5)	42 (13.9)	χ² = 2.337 (df = 1)	0.126
CSF leak	13 (5.9)	14 (4.6)	χ² = 0.405 (df = 1)	0.524
Reoperation	1 (0.5)	4 (1.3)	χ² = 1.025 (df = 1)	0.311
Readmission	10 (4.5)	24 (7.9)	χ² = 2.459 (df = 1)	0.117
Continuous variables				
Skin closure time (min), median (IQR)	7 (6-9)	14 (12-17)	U = 1,880.0; Z = −18.497	<0.001
Skin closure time (min), mean rank	119.51	366.27	-	-
Length of hospital stay (days), median (IQR)	4 (3-6)	4 (3-6)	U = 30,790.5; Z = −1.533	0.125
Length of hospital stay (days), mean rank	273.68	253.46	-	-
Inpatient cost (PKR), median (IQR)	18,000 (14,000-24,000)	18,000 (14,000-24,000)	U = 31,425.0; Z = −1.372	0.170
Inpatient cost (PKR), mean rank	270.81	255.56	-	-

Multivariable predictors of SSI

Results of the multivariable logistic regression analysis for predictors of SSI are presented in Table 3. The overall model did not reach statistical significance (Omnibus χ² = 17.382, p = 0.136) and demonstrated low explanatory power (Nagelkerke R² = 0.063), although model fit was acceptable on the Hosmer-Lemeshow test (p = 0.165). Because the overall model was not statistically significant, the individual adjusted ORs below should be interpreted with caution and regarded as exploratory rather than confirmatory. After adjustment for age, sex, comorbidities, procedure type, surgery category, implant use, skin closure time, and skin closure material, no covariate reached statistical significance as an independent predictor of SSI. This absence of statistically significant predictors should not be interpreted as evidence of no association; rather, it likely reflects the limited number of SSI events (63 events among 523 patients) and the resulting reduction in statistical power. Skin closure material was not significantly associated with SSI on adjusted analysis (Prolene vs Monocryl: AOR 0.878, 95% CI 0.224-3.443; p = 0.853); the wide confidence interval reflects the limited number of events and incomplete control of confounding.

**Table 3:** Multivariable logistic regression analysis for predictors of SSI Model fit statistics: Omnibus χ² = 17.382, p = 0.136; Nagelkerke R² = 0.063; Hosmer-Lemeshow goodness-of-fit p = 0.165. ^†^ Adjusted estimates for the malnutrition (n = 16) and combined comorbidities (n = 3) subgroups were not separately estimable in the multivariable model owing to small subgroup numbers and event sparsity (quasi-separation); these patients were retained in the model with their observed covariate pattern, but no stable adjusted ORs could be derived for these categories. SSI, surgical site infection

Variable	Adjusted OR	95% CI	p-value
Age	1.003	0.986-1.021	0.713
Sex (male vs female)	1.314	0.763-2.263	0.324
Comorbidities (overall)	-	-	0.883
None (reference)	-	-	-
Diabetes mellitus	2.487	0.274-22.591	0.418
Obesity	2.213	0.283-17.318	0.449
Malnutrition	^†^	^†^	^†^
Combined comorbidities	^†^	^†^	^†^
Procedure type (emergency vs elective)	0.594	0.318-1.110	0.102
Surgery category (spinal vs cranial)	1.143	0.340-3.845	0.829
Implant used (yes vs no)	0.376	0.108-1.306	0.124
Skin closure material (Prolene vs Monocryl)	0.878	0.224-3.443	0.853
Skin closure time (minutes)	1.016	0.881-1.171	0.829

## Discussion

In this single-center retrospective cohort study of 523 patients undergoing cranial and spinal neurosurgical procedures, we did not detect a statistically significant difference between Monocryl and Prolene in SSI or in any of the other adverse postoperative outcomes assessed. Although the SSI rate was numerically lower in the Monocryl group (9.5% vs 13.9%), this difference did not reach statistical significance, and rates of CSF leak, reoperation, and readmission were similar between groups. Importantly, the two groups were significantly imbalanced by procedure type, and the study was underpowered for the SSI comparison; the absence of a significant difference should therefore not be interpreted as evidence of equivalent safety. The single statistically significant difference favored Monocryl in terms of operative efficiency, with a markedly shorter skin closure time, although, as discussed below, this comparison is confounded by a parallel difference in closure technique. Length of hospital stay and inpatient hospitalization cost did not differ between the two groups.

The demographic and procedural profile of our cohort, with a near-equal sex distribution, a mean age in the mid-forties, a predominance of cranial over spinal procedures, and a majority of elective cases, is broadly consistent with reports from comparable high-volume tertiary neurosurgical centers [4,5,7]. The overall SSI incidence of 12.0% in our cohort lies within the upper range of reported values in mixed cranial and spinal cohorts and is consistent with rates from contemporary neurosurgical literature, including studies that have used CDC criteria for SSI ascertainment [3,5,6,19,20]. This figure is concordant with the wide range reported in the literature cited above (1-15%) and is consistent with SSI rates reported from neurosurgical and surgical cohorts in low- and middle-income settings, where rates frequently equal or exceed those of high-income centers [16,17,21,22]. In Pakistan specifically, the best available evidence indicates that SSI complicates between approximately 4% and 12.5% of procedures, and prospective single-center series from Pakistani tertiary care hospitals have reported overall SSI rates ranging from around 9% to as high as 30%, depending on case mix and wound class [21,22]. The relatively higher SSI rate compared with some high-income-country series may reflect differences in patient comorbidity, environmental factors, and the inclusion of both cranial and spinal procedures, as well as our use of broad CDC-based clinical surveillance criteria [3,4,17,19].

The trend toward a lower SSI rate with Monocryl, although not statistically significant, is consistent with prior comparative work. Previous randomized and observational studies in non-neurosurgical settings have generally reported similar SSI rates between absorbable and non-absorbable sutures [9,14,15], suggesting that suture material is unlikely to be a dominant determinant of SSI in the context of standard prophylactic antibiotic use, sterile technique, and adequate wound care. Multifactorial determinants of SSI, including patient comorbidity, operative duration, emergency status, and surgical technique, are likely to play a more substantial role than suture material alone [3,7,8,18]. Our multivariable model, which did not identify any independent predictors of SSI and demonstrated low explanatory power (Nagelkerke R² = 0.063), is consistent with this multifactorial paradigm and likely also reflects the limited statistical power of our cohort to detect modest effects.

The lack of significant differences in CSF leak, reoperation, and readmission rates between the two groups is consistent with the absence of a detectable suture-related safety difference in routine neurosurgical use, though the study was not powered to confirm equivalence for these outcomes.

The most clinically meaningful difference observed in our cohort was the substantially shorter skin closure time with Monocryl. This finding must, however, be interpreted with an important caveat: in our practice, Monocryl was predominantly placed as a continuous subcuticular suture, whereas Prolene was predominantly placed as interrupted transcutaneous sutures requiring individual knot-tying. The observed difference in closure time, therefore, reflects a combination of material and technique rather than the material in isolation, and the magnitude of the effect should be understood in that light. This finding is in keeping with prior reports from non-neurosurgical settings demonstrating that absorbable monofilament closure, particularly when performed using a continuous subcuticular technique, is faster than transcutaneous interrupted closure with non-absorbable monofilament sutures, which require knot-tying at each pass [9,15,23]. From a pragmatic standpoint, faster skin closure can translate into improved operating room efficiency, particularly in high-volume centers performing multiple craniotomies and spinal procedures per day [18]. Additional advantages of absorbable sutures that were not formally measured in our study but are well-described in the literature include the avoidance of suture removal visits, with potential cost and patient-experience benefits, and improved short-term cosmetic outcomes [9,14].

Despite the demonstrable efficiency advantage, no significant difference was observed between groups in length of hospital stay or inpatient hospitalization cost. This is unsurprising given that the postoperative length of stay in neurosurgical practice is driven principally by the underlying neurological pathology, the intraoperative course, and the development (or absence) of complications, rather than by the choice of skin closure material [3,7,17,18]. As our cost calculation was anchored to the length of stay multiplied by an average daily inpatient cost, the lack of a cost difference is internally consistent with the lack of a length-of-stay difference. We did not capture suture-acquisition costs, operating-theater time costs, or the downstream cost of avoided suture removal visits, domains in which a material-specific difference may be more readily detected. Accordingly, our null cost finding should be interpreted as referring to length-of-stay-driven inpatient hospitalization costs only and not as evidence of equivalence in total cost of care.

Limitations

This study has several limitations that affect interpretation. Most importantly, the comparison is subject to confounding by indication: Monocryl was used preferentially in elective procedures and Prolene in emergency procedures (p < 0.001), and as emergency surgery is itself an SSI risk factor, this likely inflated the apparent SSI rate in the Prolene group. Procedure type was a model covariate, but no propensity-score matching was performed, and residual confounding cannot be excluded. Second, the skin closure time comparison is confounded by technique, as Monocryl was predominantly continuous subcuticular and Prolene interrupted; the efficiency advantage cannot be separated from the technique. Third, surgeon-level clustering and experience could not be modeled, as individual surgeons were not recorded in an analyzable field. Fourth, the study was underpowered for its primary outcome (post hoc power ~33%; ~850 patients per group needed for 80% power); the non-significant model (omnibus p = 0.136) and wide confidence intervals reflect this, and the model should be regarded as exploratory. In addition, the retrospective single-center design carries risks of selection bias and limits generalizability; SSI ascertainment relied on clinical records rather than active surveillance; suture caliber and closure-technique distribution could not be reliably reconstructed; the cohort combined cranial and spinal procedures, adding heterogeneity despite covariate adjustment; and the cost analysis was confined to length-of-stay-driven inpatient cost, with cosmetic and patient-reported outcomes not assessed.

Taken together, these limitations indicate that the present findings should be regarded as hypothesis-generating rather than definitive. Our findings suggest that, in routine neurosurgical practice in a high-volume public-sector setting, Monocryl and Prolene were not associated with significantly different rates of SSI or other adverse outcomes and that Monocryl, as typically applied, was associated with faster skin closure. Larger prospective and ideally randomized comparative studies, with standardized closure technique, balanced indication-matched cohorts or propensity-score-matched analyses, surgeon-level adjustment, blinded SSI surveillance, formal cost-effectiveness analysis, and patient-reported and cosmetic outcome measures, are warranted to confirm and extend these findings.

## Conclusions

In this large single-center cohort of patients undergoing cranial and spinal neurosurgical procedures, we did not detect statistically significant differences between Monocryl and Prolene in SSI, CSF leak, reoperation, or readmission rates; however, the study was not designed or powered to demonstrate equivalence, and the comparison was limited by confounding by indication. Monocryl was associated with significantly faster skin closure, although this reflects a combined material-and-technique effect rather than the material alone. Length of hospital stay and inpatient hospitalization cost did not differ between groups. These results are hypothesis-generating and should be interpreted with caution. Adequately powered, indication-matched prospective studies, ideally randomized and with standardized closure technique, are needed before firm conclusions can be drawn about the comparative safety and efficiency of these two suture materials in neurosurgical wound closure.
